# COVID-19 Worries and Behavior Changes in Older and Younger Men and Women

**DOI:** 10.1093/geroni/igaa057.3441

**Published:** 2020-12-16

**Authors:** Sarah Barber, Hyunji Kim

**Affiliations:** Georgia State University, Atlanta, Georgia, United States

## Abstract

The case fatality rate of COVID-19 is higher among older than younger adults, and is also higher among men than women. However, worry, which is a key motivator of behavioral health changes, occurs less frequently for older than younger adults, and less frequently for men than women. Building on this, we tested whether older adults – and particularly older men -- would report the least amount of COVID-19 worry and also fewer COVID-19 behavior changes. To do so, from March 23-31, 2020, we administered an online questionnaire assessing COVID-19 perceptions, worries, and behavior changes. Participants were a convenience sample of United States residents, who were community-dwelling younger adults (18-35) or older adults (65 to 81). Analyses included 146 younger adults (68 men, 78 women) and 156 older adults (82 men, 74 women). Participants was predominately White, living in suburban/urban areas, and had completed some college. Our results showed that during the early phase of the outbreak in the United States, older adults perceived the risks of COVID-19 to be higher than did younger adults (e.g., thought COVID-19 was different than the flu). Despite this, older men were comparatively less worried about COVID-19 than their younger counterparts. Compared to the other participants, older men had also implemented the fewest behavior changes, such as wearing a mask. These tesults suggest that interventions are needed to increase COVID-19 behavior changes in older men. These results also highlight the importance of understanding emotional-responses to COVID-19, as these are predictive of their behavioral responses.

